# 518. Characterization of colistin-resistant (COL-R) *Acinetobacter baumannii calcoaceticus* complex (ABC) isolates from a recent, global Phase 3 trial (ATTACK)

**DOI:** 10.1093/ofid/ofac492.573

**Published:** 2022-12-15

**Authors:** Samir Moussa, Sarah McLeod, Alita Miller

**Affiliations:** Entasis Therapeutics, Waltham, Massachusetts; Entasis Therapeutics, Waltham, Massachusetts; Entasis Therapeutics, Waltham, Massachusetts

## Abstract

**Background:**

Due to the lack of effective therapies, last-resort agents such as colistin are more frequently being used to treat drug-resistant ABC infections. Consequently, COL-R ABC is becoming more common, with some countries such as Greece reporting rates of >50%^1^. The efficacy and safety of sulbactam-durlobactam (SUL-DUR) was recently compared to COL, both on a background of imipenem/cilastatin therapy, in patients with ABC infections, including those caused by multidrug-resistant strains. SUL-DUR was non-inferior to colistin with respect to 28-day all-cause mortality (19 vs. 32.3%). SUL-DUR therapy resulted in higher clinical cure rates and significantly improved safety compared to COL. Here, we describe COL-R ABC in ATTACK.

**Methods:**

Antibiotic susceptibility was determined by broth microdilution according to CLSI guidelines at IHMA, Inc. COL-R was defined as MIC ≥ 4 µg/ml. Next generation sequencing was performed using Nextera® libraries on an Illumina MiSeq system (San Diego, CA) at JMI Laboratories. Assembly and analysis were performed using CLC Genomics Workbench.

**Results:**

17% (30 of 175) baseline ABC isolates from m-MITT (microbiologically modified Intent-to-Treat) patients were COL-R. All were extensively drug-resistant^2^ and 26 were pan-drug resistant (PDR). Two additional ABC isolates became COL-R in patients with pneumonia who received COL therapy; neither patient survived to 28 days. Most came from 5 clinical sites: Hungary (N = 9), Russia (N = 7), Greece (N = 6), Israel (N = 4) and Turkey (N = 2). No COL-R ABC was found in China or the Americas. Sequencing analysis on selected isolates suggested sites in Hungary and Russia had clonal outbreaks, whereas others were non-clonal but closely related ( >99%). SUL-DUR was highly active *in vitro* against COL-R ABC isolates, with an MIC_50/90_ of 2/4 µg/ml. Of the 22 patients with COL-R ABC infections treated with SUL-DUR, 17 (77%) survived to 28 days with clinical and microbiological cure at test-of-cure (TOC).

**Conclusion:**

A notable number of ABC infections in ATTACK were COL-R, most of which were PDR but susceptible to SUL-DUR at ≤ 4 µg/ml. If approved, SUL-DUR could be an effective treatment for patients with these types of infections.

1. Nowak (2017) *JAC,* 72, 3277–3282

2. Magiorakis (2012) *CMI*,18, 268-81

**Disclosures:**

**Samir Moussa, PhD**, Entasis Therapeutics: Employee|Entasis Therapeutics: Stocks/Bonds **Sarah McLeod, PhD**, Entasis Therapeutics: Employee|Entasis Therapeutics: Stocks/Bonds **Alita Miller, PhD**, Entasis Therapeutics: Ownership Interest|Entasis Therapeutics: Stocks/Bonds.

